# Endemic Disease Control Agents’ perception on the fight against *Aedes aegypti* and the prevention of arbovirus infections in Brazil

**DOI:** 10.1371/journal.pntd.0007741

**Published:** 2019-10-04

**Authors:** Cíntia Pereira Donateli, Ariadne Barbosa do Nascimento Einloft, André Luiz Coutinho Junior, Rosângela Minardi Mitre Cotta, Glauce Dias da Costa

**Affiliations:** Department of Nutrition and Health, Federal University of Viçosa, Viçosa, Minas Gerais, Brazil; Northeastern University, UNITED STATES

## Abstract

**Background:**

Arboviruses pose a serious and constant threat to public health, and have demanded surveillance efforts worldwide. The prevention of arbovirus transmission depends on effective measures to control vectors and promote health. The objective of this study was to examine the factors that enhance and impair the endemic disease control agents’ field work, based on their own perspective.

**Methodology and main findings:**

In 2017, 65 ACE of seven municipalities participated in a series of seven focus groups in the Zona de Mata mesoregion (Minas Gerais, Brazil). The focus groups were organized aiming to broaden and deepen the discussion and analysis of ACE perception of their performance in relation to attributions, work processes, training, continuous education, and evaluation. All the workers, irrespective of municipality, recognize their role in disease prevention and health promotion, however they suffer from a reductionist stigma associated with their profession. Also, internal and external factors such as infrastructure, resources, administrative management, and the work process affect the quality of service delivered and job satisfaction. Practice challenges include incompatible demands such as refusal by residents and high sense of insecurity related to violence. The respondents reported that success of their activities depend on the population.

**Conclusions/Significance:**

The recurrence of epidemics demands effective vector control policies. Therefore, the performance of these professionals as regards surveillance needs to be reassessed. Public awareness and acknowledgement of the role of ACE in the identification of risk and health protection factors are indispensable for the improvement of this workforce.

## Author summary

Arboviral diseases are a growing public health concern in the world, especially in tropical areas known to be favorable for the proliferation and dispersion of the *Aedes aegypti* mosquito, the main vector of Dengue, Zika, and Chikungunya. Recurrent epidemics highlight the need for effective endemic disease control and prevention, as well as health education. Therefore, our study analyzed the perception of Endemic Disease Control Agents (ACE) about their professional performance and presented a deepened discussion about the factors that enhance and impair their work in the field. Our investigation identified four categories of factors that influence the performance of ACE: work practice and routines, challenges in the practice of the profession, work assessment, and job satisfaction. These findings reinforce ACE and professional qualification as fundamental for the prevention and control of arboviruses. Thus, measures must be put in place to promote the recognition and appreciation of these professionals. Also, the implementation of actions and allocation of resources to this sector may improve the quality of service provided, which would consequently reduce the impact of these still neglected diseases on the health and living conditions of the population.

## Introduction

Arboviruses pose a serious and constant threat to public health and have demanded surveillance efforts worldwide. According to the Global Burden of Disease Study, mortality related to Dengue in 2015 increased by 48.7% [1]. In addition, its epidemic has spread worldwide, and approximately 50% of the world´s population reside in areas with the virus [2]. Although the preventable of Dengue is 99%, elevated mortality rates have been reported worldwide [3]. A high number of co-circulating DENV serotypes in a given region is a key indicator of hyperendemicity and increased frequency of severe forms of Dengue [4].

The rising impact of arboviruses is mainly due to their high transmission, besides the capacity to adapt to new environments and hosts. These characteristics are conducive for epidemics in tropical and subtropical countries, such as Brazil [5,6]. Although Dengue is listed among the World Health Organization (WHO) priority goals, its maintenance on the list of Neglected Tropical Diseases (NTD) [7] has been questioned because of the funding and increasing scientific output related to Dengue over the past 20 years [8,9]. However, Dengue fever satisfies one of the important criteria of NTD (diseases that affect urban population in densely populated and impoverished tropical areas), thus requires effective community-based interventions, especially primary prevention [2].

The recurrence of arbovirus epidemics is related to activities that modify and transform the environment such as climate change, deforestation, urbanization, disordered occupation, displacement, and population transfer which contributes to the spread of the virus. [10]. Environmental modifications, in addition to the lack of basic sanitation, favor the transmission of pathogen to urban areas which contribute to the increased infestation and spread of *Aedes aegypti* [5,11].

Over the years, Brazil has been faced with continuous increase in the frequency and magnitude of epidemics. The period between 2010 and 2015 marked the greatest transmission of the disease in the country, with a record of six million probable cases."[12]. According to data from the Weekly Epidemiological Bulletin (Week 11), up until March 16, 2019, 229,064 probable cases of Dengue have been reported in Brazil. During this period, the state of Minas Gerais recorded the highest incidence per 100000 inhabitants in the Southeast region (261.2 cases /100000 inhabitants) [13]. This information indicates the need for health and environmental services to collaborate in order to strengthen health surveillance and ensure continuous and timely actions.

According to the National Guidelines for Health Surveillance [14], integration is mandatory for the development of comprehensive care, work protocol consistent with local reality and desired outcomes. Due to the rapid changes and diversity in global epidemiological scenarios, a broader set of players in public health surveillance activities has been shown as a recipe for success [15].

Actions related to the integration and strengthening of health surveillance are fundamental for the control and reduction of health risks. Not only do arboviruses impact infected individuals and the society, they also increase the expenditure of the Unified Health System (SUS) [16].

In the event of the co-circulation of viruses with similar symptoms, as in Brazil´s triple epidemic, Dengue, Zika, and Chikungunya that occurred in 2016, the lack of appropriate and confirmatory laboratory tests creates uncertainties about the actual epidemiological scenario being experienced [6,17,18].

The prevention of arboviruses depends on effective measures for the control of endemics and health promotion. Considering that the size of Brazil is close to that of a continent, the municipalization of health actions facilitates decision making at the municipal level as regards the best way to handle epidemics. In addition, it allows a close monitoring of vector control teams and their actions. In the case of endemic disease epidemics, regional and macro-regional entities (state) play an important role in the articulation of vector control programs [19].

In 2017, the Ministry of Health through the National Policy of Primary Health Care (PNAB) proposed the integration of Primary Health Care and Health Surveillance as an essential condition for the identification of the network of casualties and elements of the health-disease process. [14]. The 2017 PNAB made it possible to include endemic agents in Primary Health Care, collaborating with community health agents in the control of vectors and the management of the environment [14, 20–21].

However, the effectiveness of the proposed actions is undermined by challenges related to professional practice in this field. Although there are actions aimed at personal protection and mosquito control in Brazil, factors such as the lack of knowledge of the population about the actions of ACE, unpreparedness for professional practice, and precarious working conditions, e.g., infrastructure, run counter to the importance of the role played by these health environment professionals. These factors influence the recurrence of epidemics and the emergence of new arboviruses [5,22].

The fight against the emergence and re-emergence of arboviruses demands multiple intersectoral interventions and policies, and the involvement of society as being accountable for its health. Brazil must prioritize policies that invest in the availability of resources, qualification of surveillance actions, control and eradication of vectors, and restructuring of the health system, especially with regard to decisions that directly impact the population [22].

In order to understand the context associated with cyclical epidemics of arboviruses in the Zona da Mata region of Minas Gerais, Brazil, the objective of this study was to examine the factors that enhance and impair the endemic disease control agents’ field work, based on their own perspective.

## Methodology

### Overview of study methods

This study is a qualitative study which employed the focus group technique. The sampling technique of this study was non-probabilistic (convenience) sampling involving 65 ACE aged between 23 and 57 years. Via focus group discussions, we were able to facilitate in-depth discussions focused on the perception of ACE regarding their own performance. By using focus groups, we were able to generate evidence to support opinion assessment and interact with professionals of the Unified Health System (SUS). In order to clarify the findings, it was essential to understand the actions of the agent in the fight against *Aedes aegypti* and prevention of arboviral infections in Brazil.

In this research, the concept of performance encompasses not only the attributions of ACE, but also broadly covers job qualification, evaluation and professional valorization.

### Study setting and participants

The state of Minas Gerais is divided by the Brazilian Institute of Geography and Statistics (IBGE) into twelve mesoregions, covering 66 microregions. The Zona da Mata mesoregion of Minas Gerais consists of seven geographic microregions: Ponte Nova, Manhuaçu, Viçosa, Muriaé, Ubá, Juiz de Fora, Cataguases. In the last census, the largest municipality had a population of 57,390 inhabitants and population density of 121.94 inhabitants/km^2^ while the largest city had a population of 516,247 inhabitants and a demographic density of 359.59 inhabitants / km^2^ [23].

This study is a subset of the project "Health Surveillance: evaluation of disease prevention and health promotion practices in the Zona da Mata region of Minas Gerais". The first study of this project evaluated and classified health surveillance in the Zona da Mata region, Minas Gerais, as intermediate, indicating its fragmented performance in the reorganization of health practices. [24]. After this stage, the need arose to analyze ACE perception of their professional performance, among workers that did not participate in the previous study. The initial quantitative phase consisted of the application of a structured questionnaire, where all ACE from the seven municipalities (n = 321) participated. After the quantitative analysis, we felt the need to deepen discussions on different subjects highlighted by ACE. For this purpose, we chose the focus group method as propitious for the exploration of these subjects.

The present study employed a non-probabilistic sampling method, which involves the selection of ACE according to interest and willingness to participate, following a strict focus group methodology based on eight to ten ACE per municipality. The main fieldwork in this profession includes carrying out treatment with insecticides and monitoring of surroundings with the objective of reducing mosquito breeding sites, especially during home visits as well as strategic areas such as abandoned lands and construction sites.

The focus groups were held at the workplaces of ACE located in the premises of the environmental surveillance and zoonoses department in the municipalities of the Zona da Mata region of Minas Gerais: Cataguases, Juiz de Fora, Manhuaçu, Muriaé, Ponte Nova, Ubá, and Viçosa. Eligibility was based on the following criteria: male or female 1) willing and able to provide informed consent, 2) professional field practice experience, and 3) willing and able to complete a 60-minute focus group.

### Development of the focus groups

The ACE were informed about the objectives and goals of the study and their participation before receiving the informed consent forms. All participants signed the informed consent form prior to any study activity.

In each municipality, one focus group was formed, totaling seven focus groups for the seven included municipalities. Data collection took place in the second semester of 2017. The data collection process involved the participation of a researcher/moderator, an observer and ACE. The moderator facilitated the focus group discussion using a focus group guide developed by the research team and to capture the perception of the participants about the topic "Performance of endemic disease control agents (ACE) in the Zona da Mata mesoregion of Minas Gerais".

The focus group guide had five questions to explore the main themes of interest: 1) How do you perceive the role of the endemic disease control agent in health promotion and disease prevention? 2) Are there inherent challenges in the practice of the profession? If yes, what are they? 3) Are there difficulties related to the training process for fieldwork? If yes, what are they? 4) How does the assessment of the agent’s field work occur? and 5) What can be done to improve the work of endemic disease control agents so that they achieve job satisfaction?

The researcher/moderator allowed a free-flowing and directed discussion during the focus group meetings, which kept the discussion within the scope of the research objectives.

### Qualitative analysis

All focus group discussions were digitally recorded and fully transcribed. Participants were randomly coded (from ACE1 to ACE65) to ensure anonymity. All the transcriptions underwent qualitative analysis, where, the citations were classified into concepts by the thematic analysis method using 0.7 alpha 2 IRAMUTEQ (Interface de R pour lês Analyses Multidimensionnelles de Textes et de Questionnaires).

Textual contents were analyzed using descending hierarchical classification. It groups and organizes the corpus graphically according to the frequency and similarity of words. In the analysis, only words with frequency greater than or equal to mean frequency, chi-square (x^2^) greater than or equal to 3.84 and p ≤0.05 were considered.

After the transcription and reading of the filed material, an analytical model was built with four categories, which corresponded to the word classes generated by IRAMUTEQ. In this study, we chose post-collection analytical categories because they are more specific and concrete, coupled with lexical criteria [25]. The classes found synthesized the study findings, thus they do not necessarily have to correlate with the number of guide questions. The analysis of the textual corpus followed the sequential stages of content analysis proposed by Minayo (2014) [25] and Bardin (2004) [26]: 1) pre-analytical phase; 2) the exploitation of the material; and 3) treatment of results, inference, and interpretation.

As a summary, we present a SWOT (Strengths, Weaknesses, Opportunities and Threats) analysis of the work process of ACE.

### Ethical approval

All the participants signed an informed consent form and the study was approved by the Human Research Ethics Committee of the Federal University of Viçosa (UFV) under number 1447272, respecting all ethical procedures. The study is part of a larger project entitled "Health Surveillance: evaluation of disease prevention and health promotion practices in the Zona da Mata region of Minas Gerais"

## Results

### Characteristics of participants

Among the 65 participants of the seven focus groups, 53.85% were female, with greater participants within the age group of 31 to 35 years (26.15%). The variable “job position” was investigated, since many ACE agents also hold area supervisor positions, thus some of the agents are subordinate to them. This control was necessary, as this situation could influence the statements provided by ACE, however this problem did not occur. All participants were given equal opportunity to express their opinions individually and collectively as a group (Table 1).

**Table 1:** Profile and Characteristics of Endemic Disease Control Agents (ACE) of the Zona Mata Mesoregion, Minas Gerais, Brazil, 2018.

Variable	N	%
**Sex**		
Male	30	46.15
Female	35	53.85
**Age**		
< 25 years	04	6.15
25–35 years	32	49.23
36–45 years	19	29.23
46–55 years	08	12.31
> 55 years	02	3.08
**Position**		
Field agent	60	92.31
Area supervisor	05	7.69

### Clarifying ACE performance

The corpus consists of 2,030 text segments (TS), being 1,883 TS capable of use, corresponding to 92.76% of the total corpus.

**Fig 1:**
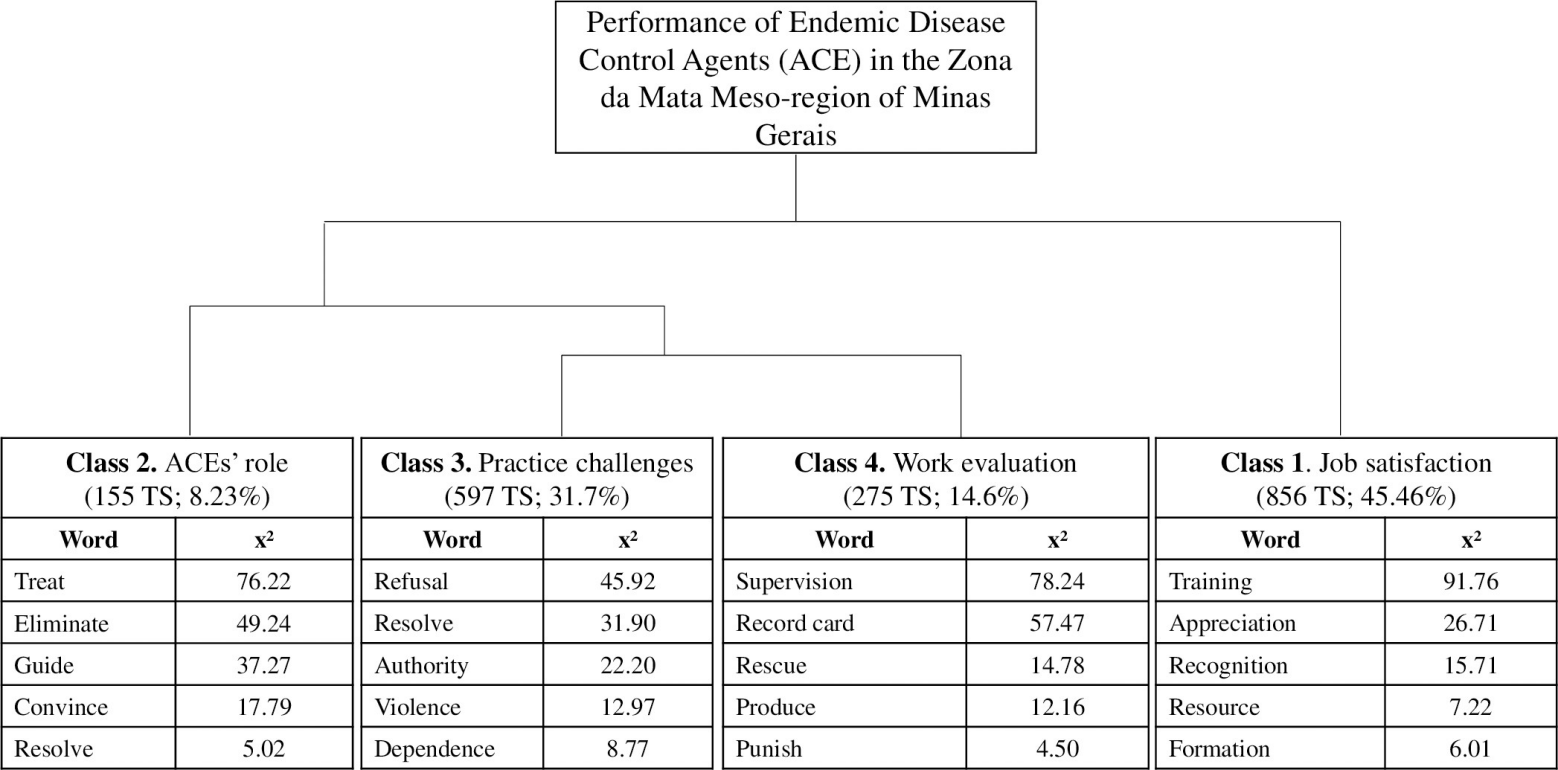
Dendrogram of the Descending Hierarchical Classification (DHC) containing partitions and contents of the corpus which represents the conceptions of the Endemic Disease Control Agents (ACE) about their performance. (TS) are the text segments, and (x^2^) is the chi-square value. All the words presented had statistical significance (p <0.05).

Four main themes emerged from the focus groups: the role of ACE, practice challenges, fieldwork assessment, and job satisfaction. They are summarized in the dendrogram (Fig 1) and presented in the main discussion topics.

### Role of endemic disease control agent

The participants reported the function of ACE in both disease prevention and health promotion.

"*We perceive the role of our work in health promotion and disease prevention as the front line of endemic control*, *to me it's like being the lifeguard of the community*" (ACE6).

The prevention activities commonly reported were treatment and elimination.

"*The role of ACE agents is to work with prevention*. *The main priority is to eliminate vector*, *at least as regards our job committed to households*"(ACE14).

"*In the households*, *we treat places that store water before the mosquito stage*, *eliminating dengue as well*" (ACE28).

In addition, the participants pointed out promotion of health as being the function of ACE and considered ACE a guiding agent of the population. The ACE reinforce the importance of this guidance role, but still have difficulties in conducting routine interventions, since in some cases they need to convince residents about the importance of home inspection for the elimination of mosquito foci in order to have access to the houses and perform related services.

"*Here we have a project called ‘Positive Aedes’ aimed to guide the resident to keep house and yard clean*" (ACE2).

In this sense, the ACE reported carrying out health education actions, including in schools:

"*We have an educational task in schools*, *through lectures*, *we show mosquito breeding sites*, *the larvae*. *It's an important job as well*. *This work is not only done in schools*, *we go to health care units*, *we gather residents of neighborhoods for disease prevention*, *asking everyone to collaborate with us* "(ACE30).

"*Children are our greatest hope*. *After we carry out activities with them*, *on returning home*, *they guide their parents to correct what was shown as being wrong* "(ACE44).

Another issue addressed is the need for solution to problems highlighted, which is sometimes not possible due to the lack of resources, for example, the availability of screens, which would solve the problem concerning uncovered water storage tanks. Moreover, too often, the resident can not afford the material required, thus it is neither possible to demand the item nor carry out the treatment. In addition, ACE face problems with sites that have recurrent outbreaks.

"*Often our guidance most of the time*, *which I think is what weighs most among us*, *does not solve the problem*, *if it did*, *it would not take on such proportions*. *I went to a place*, *I did the complete control intervention*, *correctly*, *and today I got there and everything was the same as before* "(ACE51).

"*So*, *we still find houses with asbestos water tanks*, *uncovered tanks*, *but we can not solve these problems*. *There are no resources for this*, *even less ask the resident* "(ACE16).

Some ACE pointed out the ineffectiveness of emergency actions carried out by them, in the short and in the long term.

"*I think in many cases we are not effective*. *We go to the house*, *do the control intervention and give guidance to the resident*. *In the next cycle*, *we return and the problem continues*. *We have no conditions to work in the long term*. *Our work ends up not being effective* "(ACE35).

### Practice challenges

ACE reported many challenges, with the most frequent being refusal on the parts of residents to let them in their houses, followed by low effectiveness of the actions, lack of authority, and constant exposure to violence at work.

The reception of the agents by residents goes through several phases, but the refusal of entry prevents the carrying out of their work. According to the agents, the main trigger for this refusal is the lack of credibility and the high turnover of professionals in the target areas, so residents are not familiar with them and, consequently, do not create any relation. These challenges imply even more refusals, in addition to other impediments in this process such as criminality and violence, which produce a sense of collective insecurity.

"*There are many situations in which not anyone can go to a certain area because it is dangerous*" (ACE62).

"*Another situation that happens is when you have to visit a higher-class neighborhood and residents refuse entry because they doubt that you are a municipal agent*, *and often we can not even argue because we have no identification and no uniform*" (ACE57).

These aforementioned challenges make the ACE agent feel dependent on the resident to do their job. Thus, they believe that the use of force and authority, through collaboration with the Brazilian army in the municipalities, will change this situation:

"Similar to *the support we had with the aid of the army*, *those who refused our entry were even frightened when we returned with a soldier*, *who was actually there just to accompany us*" (ACE23).

The lack of clarity in the work process places the ACE in an unfavorable condition, not being able to solve many problems of the population. This dependence makes them put blame on residents. However, this surveillance process needs to be viewed as a partnership, where agents and residents are key players.

"*There is a lot that we can not solve*, *for example garbage*, *there are residents who discard it anywhere*, *let it accumulate and do not care*. *It's very sad when we can not do anything*. *We depend on the community to find solution* "(ACE58).

"*There are people who think fines would solve it*, *but I do not think so*. *I think it would get worse*. *The good thing would be if our notifications were decisive*, *that would be great* "(ACE49).

### Evaluation

The evaluation of the work process of ACE is carried out in the conventional format. The supervision of their house inspection occurs through direct control, when the supervisor follows the agent during a visit, or indirect supervision, which occurs after the agent’s intervention, where the supervisor inquires about the performance of ACE from the resident. In addition, there is control by record sheets, sheets placed in the house to control visits, bulletins, and reports sent to the supervisors/managers for evaluation and monitoring of activities. However, ACE considered this evaluation method inefficient:

"*The criteria evaluated are about the working method*, *the approach with the resident and production*, *but unfortunately we do not get feedback*" (ACE32).

The biggest complaint is the lack of feedback. There is a conflicting hierarchical relationship with authoritarian attitudes that further undermines the work of the ACE in the field.

"*Unfortunately there is no feedback*. *We*, *field agents feel we are orphans*, *that's the word*, *orphan*, *because we do not get any feedback*. *Our supervisors do not tell us what our evaluation was like*, *we do not know what is going on*. *The only feedback we get is that we are not meeting the goals* "(ACE65).

"*The importance of feedback would be to relay information to the residents*. *We do not know how the disease situation in the city is*. *The resident asks and we do not know how to answer about our own job*. *In the epidemic*, *we did not even know the number of cases* "(ACE3).

Complaints about the evaluation also include productivity demands, which often disregards the particularities of a given locality, the days and times when the intervention needs to be performed, and insufficient number of professionals to cover the whole territory.

"*We can not produce as much as we need for that day*. *Our lunch schedule is the same as the resident*, *so we will never meet someone at home*. *Thus*, *we are producing less because of this* "(ACE25).

"*We work for quantity*, *they do not want to know about quality* " (ACE59).

### Job satisfaction

Some reports help us draw conclusions about the contribution of recognition and appreciation toward job satisfaction, given the feeling of demotivation and uselessness pointed out by the agents.

"*The residents think we are only there for Dengue*. *They see us only as agents of Dengue* "(ACE41).

"*We see this need for appreciation and commitment to offer us training*, *in addition to disseminating the work of the endemic agent*, *because many times our training is very limited*" (ACE11).

The feeling of inferiority when people label ACE as ‘dengue boys’ (reported by agents) generates an urgent need to change this profile. Among the issues faced, they point out the need for a vocational course with specific work-related training because they feel unprepared.

"*We should have a training course*, *with a specific methodology to train endemic agents*. *Nowadays*, *we learn the practice with other agents in the field* "(ACE13).

Qualification and continuous training would promote the recognition of ACE agents, a reality almost non-existent to them.

"*There was an outbreak of Dengue in the neighboring city*, *so we did a lot of work here and we had almost no cases that year*, *but for the coordinators there was no Dengue because it did not rain*. *Thus*, *our work is useless* "(ACE40).

The lack of resources heightens the feeling of undervaluation and lack of recognition

"*When we go to the field*, *we have to rely on the goodwill of the resident to let us use the bathroom*, *drink water*. *If they see us under a shade*, *resting a little because of the sun*, *they say at once that we do nothing* "(ACE1).

**Fig 2:**
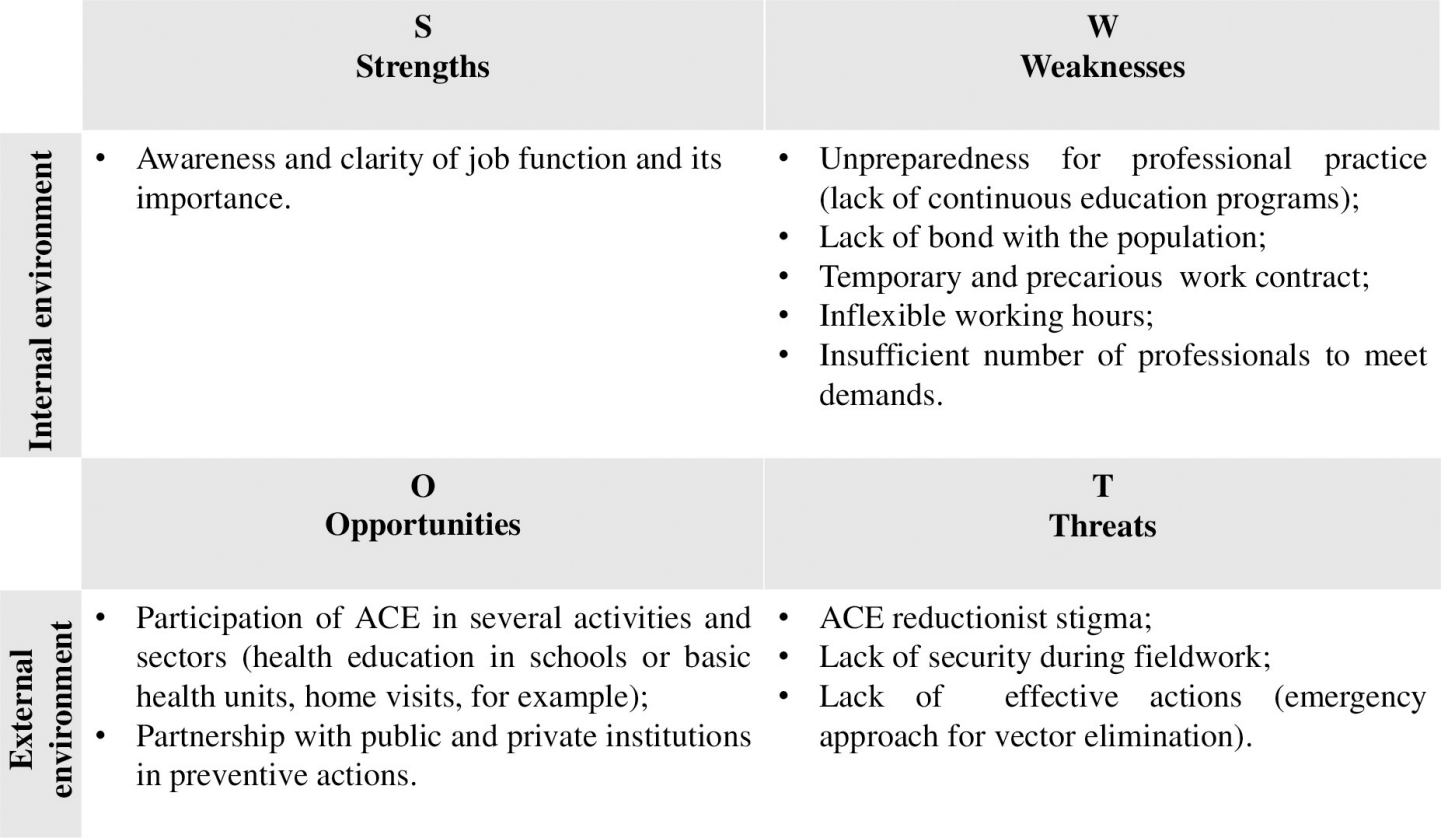
SWOT analysis.

We present a SWOT analysis of the work of ACE, highlighting the Strengths and Weaknesses (internal environment) and the Opportunities and Threats (external environment) (Fig 2). It was found that the Weaknesses and Threats were predominant, demonstrating that the work process of ACE presents some degree of vulnerability, and the Weaknesses may amplify the Threats.

## Discussion

ACE agents play a critical role in the organization of community health work. The perception of ACE about the community is related to the lack of education and knowledge of residents on how to keep their property safe from mosquito foci. Blaming the population for the low efficiency in solving problems identified and low effectiveness of actions ruins the relations between the subjects. These findings corroborate with studies that address the importance of education, preventive practices and co-responsibility of the population and public authorities for *Aedes aegypti* control [27,28]. It requires collective adherence on the part of all the players involved to change the health profile of the country and advance in the reduction of health inequalities, considering empowerment and education as precursors of this change.

The endemic disease control agents are in direct contact with the community and environment and their work involves multiple risks. The perception of danger was reported for contact with insecticides and pesticides, falls from heights, and violence. Another study [29] portrays these same risks related to health, in its expanded concept, and the work of ACE, emphasizing the interference of social interaction with urban violence and its various contours such as drug trafficking, physical and verbal aggression during the visits, and the health repercussions in the lives of these workers.

Moreover, the ACE feel there is a lack of regulation regarding their autonomy and recognition of their role in the health of the population. Actions such as the dissemination of their work in communication channels would increase the credibility and acceptance by the residents, resulting in fewer refusals and higher productivity. Recognition by the population is not enough, managers and coordinators need to give ACE a listening ear and include them in the decision-making processes to promote efficiency and problem solving skills.

Therefore, in this sector, communication and dialogue between both parties is indispensable. Bulletins or reports should be discussed and evaluated, most importantly there must be a feedback system that conveys failures and possible improvements that can be made. In this sense, there is room to promote meetings where employees are aware of their weaknesses and strength. Also, both parties can share their experiences and daily practices, all of which can improve the quality of service delivered [30].

The results of this research suggest that the work of ACE is not recognized; moreover, these workers suffer from insecurity, lack of resources and training. It can therefore be inferred from the findings that the work of ACE is not properly evaluated. In the same line of research, Donateli and collaborators (2017) [24] designed a logical model of health surveillance. In this model, hiring and training of human resources were considered managerial functions, mainly of the health surveillance coordinator. As previously mentioned, this does not correspond to reality. The most common situation is training programs conducted by an experienced ACE within the work environment. Despite having experience, he/she may not have the appropriate skills to perform these trainings, for instance, effectively convey to beginners and inexperienced colleagues how to properly perform tasks related to the job.

Studies that evaluated the knowledge of health professionals or high school students about arboviruses identified gaps [31,32]. According to the studies, the professionals need to be continuously trained and qualified for the control and management of arboviruses. According to Corrin, et al. (2017) the lack of knowledge is a barrier to individual protective attitudes and joint interventions against the mosquito [33]. Thus, the actions of ACE need to be strengthened through communication and dissemination. In the study of Fritzell et al. (2016) [31], school intervention, television and social media were cited as adequate communication tools to promote public health messages.

The triple arbovirus epidemic in Brazil resumed the debate on the failure of local actions to eradicate *Aedes aegypti*, significantly blamed on limited capacity of municipal planning and management of health surveillance [10,18].

Although the decentralization of disease control has allowed the municipality to expand its actions, the control and prevention of infectious diseases heavily relies on mandatory notification issued in the Notifiable Disease Surveillance System (SINAN) [29]. Thus, work in this field depends on constant case notification and investigation, data transmission to other levels, and the construction of databases and information systems [34]. The compartmentalization of surveillance has impaired necessary integration with networks within the Unified Health System (SUS), which results in limited response to local needs and problems [9,24].

Vector control strategies of the government have been based on top-down approaches coupled with vertical programs [35] and chemical-dependent model for the control of *Aedes aegypti* [36], despite report indicates the contrary: isolated vector control initiatives do not promote behavioral changes [37,38].

ACE are recruited through a civil service examination and/or proof of qualification. The contract of ACE is determined by the period established to meet a temporary demand [39]. The temporal nature of the job implies high turnover of the professionals, which undermines the bond of ACE with the community. In addition, although ACE are residents of the city in which they work, they are not always allocated to their neighborhoods. By simply allocating ACE to areas they reside, the sense of security of both ACE and residents will increase, reducing resident refusals, and creating a stronger bond between both parties.

Employee training is highlighted as fundamental for an integrated and effective work. Training programs should be continuous, and structured to improve employee skills, most importantly, cover topics relevant to the practice of the profession. According to a study conducted in Belo Horizonte, the capital city of Minas Gerais [40], factors such as the lack of investment in professional training, low salary, precarious employment relationship, and poor working conditions largely contribute to the fragile professional identity of ACE. This scenario is equally alarming as the emergence and reemergence of arboviruses that have plagued the country for years.

It emphasizes that health surveillance service and actions need to undergo a complete reorganization, with the main priority being the recognition of the functions of ACE in direct and continuous contact with the community, land inspection and vulnerability mapping. Therefore, ACE integrates the community and health service, which is important for the implementation of effective arbovirus prevention and control actions that require multilevel engagement. They are also capable of having a broader perspective of health determinants as well as environmental related-health risks, both in the individual and collective sense.

### Limitations

The content of the article should be analyzed based on some limitations. Although the exploratory and comprehensive design of the study allowed a more profound analysis of the complexity of the phenomenon studied (fieldwork of ACE), the generalization of the results to other context, should be done with caution, moreover the results should not be extrapolated. The sampling technique employed in this study was non-probabilistic (convenience) sampling. The ACE who were present on the data collection day and who accepted to participate in the focus group were included in the study. Factors related to subjectivity and personalities made it difficult to extract information about categories and characteristics using qualitative data analysis, however this limitation was minimized by employing the Iramuteq software.

### Conclusion

Future challenges in relation to epidemics, neglected diseases, and vector control are innumerous. Thus, an in-depth knowledge of the actions of ACE provides an important insight into the experiences and perceptions of this workforce in the face of the epidemiological scenario in Brazil characterized by disease threats and increased demands. The present study shows the inadequate analysis of the work process of ACE. Our findings reveal that ACE suffer from the lack of recognition, low efficiency of actions, and devaluation.

Clearly, the society and government need to restructure their approach to urban health problems. The recurrence of epidemics indicates that public policies have not been effective as they should be. Therefore, understanding and acknowledging the fundamental role of ACE in the identification of risk factors and health protection could contribute to the effective eradication and control of epidemics. Approaches such as practical solution to challenges, valorization of this workforce, employee training, establishment of reliable assessments methods and cost-effective interventions do not only improve service quality but also contribute to the fight against health problems, and consequent improvement of the health status of the population.

In view of the above, more studies are needed to design and evaluate the professional performance of ACE in other locations and populations. We also suggest that studies use triangulation, in order to consolidate the conclusions of the phenomenon studied as well as identify similar or additional factors related to this subject of extreme relevance to public health.
